# The Influence of Surgical Mask on Heart Rate, Muscle Saturation of Oxygen, and Hemoglobin during Whole-Body Vibration Exercise

**DOI:** 10.1155/2022/3958554

**Published:** 2022-11-22

**Authors:** Mª. Dolores Apolo-Arenas, Pablo Tomas-Carus, Pablo Galan-Lopez, Jorge Navarro Escribano, Beatriz Carvalho, Alejandro Caña-Pino, Jose Alberto Parraca

**Affiliations:** ^1^Departamento Terapéutica Médico Quirúrgica, Facultad de Medicina, Universidad de Extremadura, 06006 Badajoz, Spain; ^2^Departamento de Desporto e Saúde, Escola de Saúde e Desenvolvimento Humano, Universidade de Évora, 7004-516 Évora, Portugal; ^3^Comprehensive Health Research Centre (CHRC), Universidade de Évora, 7004-516 Évora, Portugal; ^4^Department of Communication and Education, Universidad Loyola Andalucía, 41704 Dos Hermanas (Sevilla), Spain

## Abstract

**Background:**

Whole-body vibration (WBV) is a safe and effective exercise system that affects muscle oxygen through several physiological processes, although its effects on different protocols are still unclear. Unfortunately, the COVID-19 pandemic has generated various health problems and controversy or confusion on its possible adverse consequences and impact on performance when wearing a mask during the practice of physical exercise.

**Aim:**

To analyze the acute effects of WBV exercise in muscle oxygen variables during different intervention phases with or without a surgical mask and compare protocols that differ in the order of vibration frequencies.

**Methods:**

Forty-seven healthy students participated in WBV training. They were randomly assigned to use or not use a mask between the three intervention groups: group A (8, 12.6, and 20 Hz), group B (12.6, 20, and 8 Hz), and group C (20, 8, and 12.6 Hz). Besides the 3 WBV moments, the intervention had a baseline moment, two rest time and a recovery moment. During the whole intervention, the heart rate (HR), muscle oxygen saturation (SatO_2_), oxyhemoglobin (O_2_Hb), and deoxyhemoglobin (HHb) were registered.

**Results:**

There were no significant differences between the mask use and not use groups. Significant differences were found between the variables during the seven intervention moments and between intervention groups (A, B, or C).

**Conclusion:**

HR, SatO_2_, and Hb were not influenced by the use of a surgical mask, but they reacted differently through the different moments and were sensitive to vibration frequencies and respective order.

## 1. Introduction

Vibration is a mechanical stimulus characterised by an oscillatory motion in which energy is transmitted from the actuator (the vibrating device) to the human body [1]. During sporting activities, our body interacts with the environment and experiences external forces that induce vibrations and oscillations in body tissues [2].

Nevertheless, the literature clearly showed that exercises on a vibrating platform are safe, feasible, and well-tolerated by patients with different disorders, with associated force and muscular power increase [3]. Similarly, this type of therapy is increasingly entering the clinical and biomedical field, being currently a quite positive complementary treatment in physiotherapy [3]. In particular, the effects of the WBV had been studied in healthy subjects, using vibrational platforms, especially designed, which produces sinusoid vibrations [4].

On this line, mechanical oscillations (30-50 Hz) in the body stimulate various biological systems [5]. The oscillating and vibrating platform produces rapid and short-term changes in the length of the muscle-tendon complex [6]. These disturbances are detected by sensory receptors that modulate muscle stiffness through reflex muscle activity and attempt to dampen vibratory waves [7]. Additionally, mechanical stimulation in the form of vibrations of the human body is an effective way to improve muscle strength, body balance, and the mechanical component of bones [8–10]. Regarding the short-term effects of WBV exercise on muscle performance, increases have been shown in isometric leg extension strength in female volleyball players [11].

Further on, the neuromuscular response to WBV exercise differs on the type, frequency, amplitude, and duration of the oscillator stimulus, as well as on the position of the patient's body on the vibrating platform [12]. Among these factors, the frequency of vibration appears to play an essential role in the magnitude of the neuromuscular response. Considering the numerous combinations of amplitudes and frequencies possible with current technology, it is clear that there is a wide variety of WBV exercise protocols used in humans [13].

Moreover, additional mechanical stimulation of WBVs is also known to lead to increased acute energy turnover indicated by increased oxygen uptake. This effect becomes more robust with increasing frequency, amplitude, and additional training load [14]. Also, changes in peripheral blood flow resulting from the application of WBVs could indicate a possible mechanism of action for treatment with WBV [15]. If WBV exercise increases blood flow (oxygenation), it could be used as a therapeutic intervention when the clinical goal is to increase blood flow or muscle oxygenation [16]. However, it is crucial to measure oxygen saturation and to know the behaviour of oxygen levels in the blood during physical exercise; body cells can be damaged if values are low [17].

Furthermore, muscle oxygen saturation index (SatO_2_), oxyhemoglobin (O_2_Hb), and deoxyhemoglobin (HHb) are measurements used to determine the balance between oxygen (O_2_) supply in a specific muscle that can be indirectly obtained through a noninvasive technique, the near-infrared spectroscopy (NIRS) [10, 18–20].

In the context of the COVID-19 pandemic, the use of the surgical mask during sports has generated controversy or confusion about its possible adverse effects on performance. Several studies have analyzed the physiological effect of facemasks during exercise without obtaining conclusive results [21, 22]. These include hypotheses such as the oxygen uptake being compromised and the air retention level in the mask will increase carbon dioxide rebreathing causing hypercapnic hypoxia, where an increased arterial carbon dioxide displaces oxygen from hemoglobin [23].

Given the above, the primary study objective is to analyze the acute effects of WBV exercise on muscle oxygen variables during the different phases of the intervention with or without using a surgical mask and compare protocols that differ in the order of vibration frequencies. Further, we pretend to (a) analyze the effect of the use of the mask on HR, SatO_2_, O_2_Hb, and HHb during the WBV exercise in different phases of the intervention and random intensities, (b) analyze the behaviour of tissue oxygenation in the different phases of the intervention, and (c) check if this behaviour is sensitive to different vibration frequencies order.

We hypothesize that (i) the use of disposable surgical masks influences tissue oxygenation during the WBV training compared to not using disposable masks; (ii) the use of a disposable surgical mask during the WBV training influences HR; (iii) there are differences in tissue oxygenation between exercise and rest periods during the WBV training; and (iv) the differences in tissue oxygenation between exercise and rest period's during the WBV training are sensitive to the vibration frequencies order.

## 2. Methods

### 2.1. Ethical Considerations

The Ethics Commission for Scientific Research in the Areas of Human Health and Well-Being of the University of Évora approved this experimental study (registration number 19027) that followed all the ethical guidelines according to the Declaration of Helsinki. All participants signed an informed consent form; each contained the objectives of the study, procedures used, duration, results and benefits expected from their participation in the study, and some notes on the confidentiality of the data obtained.

### 2.2. Subjects

**Figure 1:**
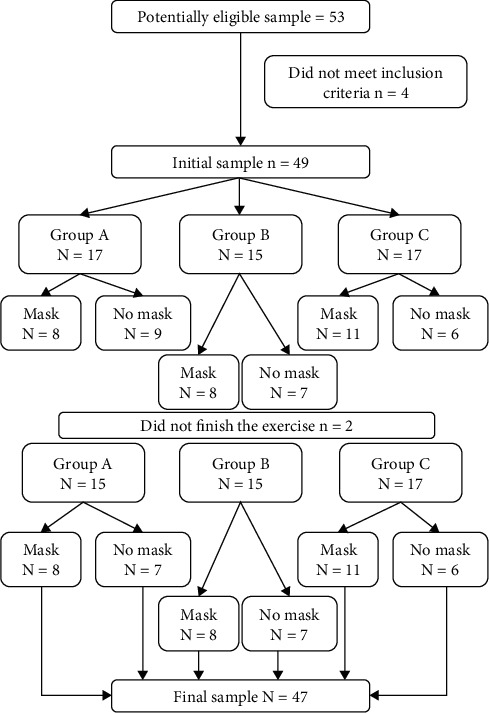


Forty-seven of the fifty-three recruited students of the Sports and Health Department, University of Évora, completed the intervention. As shown in the Intervention Flow Diagram (Figure 1), four of the students recruited did not meet the inclusion criteria, while the other two did not participate in the intervention. Specifically, the following inclusion criteria were applied: (a) age between 18 and 28 years old, (b) being healthy, (c) no musculoskeletal injuries, (d) no previous experience in WBV training, and (e) practice less than 45 minutes of physical activity per week. Exclusion criteria included (a) major injuries, (b) headache, (c) herniated disc, (d) inflammatory rheumatic disease, (e) pregnancy, and (f) doing physical activity 12 to 24 hours prior to the intervention. The final sample comprised 47 students (16 female and 31 male), with ages between 18 and 28 years old (19.9 ± 2.0 years), a weight of 67.8 kg ( ± 11.5) and a height of 171.0 ( ± 11.0 cm).

### 2.3. Experimental Protocol

In collaboration with the University of Extremadura and the University of Évora, we performed an experimental study to analyze the effects of body vibratory exercise. This cross-sectional and randomized study had a participation of 47 healthy students; 31 of them were men and 16 women, and a total of 27 subjects performed the intervention with a mask, while 20 performed it without a mask. All participants performed an intervention on a vibrating platform. They were randomized into three groups to make them homogeneous. In each group, the research team applied three vibration frequencies in a different order and for one minute for each frequency, separated by one minute of rest; these frequencies were group A (8-12.6-20 Hz), group B (12.6-20-8 Hz), group C (20-8-12.6 Hz). In each of these groups, participants were further divided into those who wore masks and those who did not.

### 2.4. Materials and Methods

The instruments used during the intervention and data collection were a heart rate meter, a stopwatch, a muscle oxygen monitor, and a vibrating platform. The HR was collected using an H10 chest strap (Polar Inc., Kempele, Finland), placed at the xiphoid level, monitored by Polar Vantage M (Polar Inc., Kempele, Finland), and placed on the participant's wrist.

For the oxygen-related variables: muscle oxygen saturation (SatO_2_), oxyhemoglobin (O_2_Hb), and deoxyhemoglobin (HHb), we used the muscle oxygen monitor Moxy-3 (Moxy, Fortiori Design LLC, Minnesota, USA). The research team placed the monitor in the medial portion of the vastus lateralis, measuring its origin (greater trochanter of the lateral aspect of the femur) and insertion (tibial tuberosity and patellar ligament (patellar tendon)). The software used to monitor and examine this variable was the GoldenCheetah System v.3.5 (http://www.goldencheetah.org/).

To keep track of the time of the test, we used a chronometer; and following the time sets, we gave instructions to each participant during the trial. The vibration platform used for this study was a lateral oscillation platform divided by a median axis, Galileo Fitness (Novotec Medical GmbH, Germany). This device emits sinusoidal vibration with a frequency range between 5 and 30 Hz and an amplitude between 0 and 5 mm.

The surgical mask or the term “surgical mask” in this study refers to the usually blue disposable face masks certified to medical standards [24] that used to only be seen among healthcare professionals and which use has currently spread to the rest of the population. Being one of the most common face masks used by standard population since the COVID-19 pandemic, it was relevant for this study to use such masks and evaluate the physiological effects they have. Furthermore, previous research comparing physiological differences between the use and not use of surgical masks in physical tests used this same type of mask [25, 26].

### 2.5. Procedure and Protocol

The procedures used during the experiment were the following: first, the researcher gave the group oral information on the work's purposes and the inclusion and exclusion criteria. Second, following the requirements previously described, the researchers recruited 49 volunteers for participation. Third, the participants signed the informed consent. Fourth, the researchers evaluated participants' health and activity status, including physical activity practice, age, weight, and height, using a sociodemographic questionnaire. Fifth, the researchers placed the heart rate meter and muscle oxygen monitor, using the aforementioned placement. And lastly, start of the intervention.

The entire sample is subjected to an intervention on the Galileo Fitness platform (Novotec Medical GmbH, Germany) using three different vibration frequencies (8 Hz, 12.6 Hz, and 20 Hz), randomized from one subject to another: A: 8 Hz, 12.6 Hz, and 20 Hz; B: 12.6 Hz, 20 Hz, and 8 Hz; and C: 20 Hz, 8 Hz, and 12.6 Hz. Each participant performed one minute of each frequency interval within each group with one minute of rest. In each minute of the WBV, the participants had to maintain a bipedal posture with their hands holding on to the bars of the vibrating platform and maintaining a 60° knee flexion.

The researchers also evaluated the basal levels three minutes before and three minutes after the test through physiological parameter monitorization (Table 1). All collaborating researchers from the University of Évora and the University of Extremadura underwent a training and a learning process to collect the physiological parameters. The data collection occurred at the intervention site, the University of Évora Pavilion.

### 2.6. Data Analysis

The variables collected during the study were four demographic variables (age, sex, weight, and height), the heart rate (HR, measured in bpm), and three oxygen-related measures: muscle oxygen saturation (SatO_2_, measured in percentage), oxyhemoglobin (oxygenated hemoglobin or O_2_Hb, measured in g/dL), and deoxyhemoglobin (deoxygenated hemoglobin HHb, measured in g/dL).

We used Excel (version 2016) and jamovi 1.6.6 programs for data analysis. First, we performed the Shapiro-Wilk normality test. Additionally, we performed a sample descriptive analysis, categorized by mask use: with or without, and intervention type: A, B, or C. Next, we compared means for each of the variables measured and categorized by the mask use and the type of intervention at the different moments of the intervention. For this comparison, we performed a one-way ANOVA. Afterwards, we performed repeated means ANOVA comparing related sample means of each variable measured on the different intervention moments.

Subsequently, the effect size was analyzed by calculating Cohen's d. We compared effect size using eta squared means (*η*p2), concerning that proposed by Cohen, being small (TE ≤ 0.06), medium (0.06 < TE ≤ 0.14), and large (TE > 0.14). To calculate the magnitude of the effect, as well as the limits (upper and lower), the standardized mean differences and the respective 95% confidence intervals were calculated, being <0.2 = trivial; 0.6 = small; 1.20 = moderate; 2.0 = large; and >2.0 = very large. At last, with an ANOVA pairwise comparison, we compared the heart rate, muscle oxygen saturation, hemoglobin, oxyhemoglobin, and deoxyhemoglobin between the three groups in different intervention moments.

## 3. Results

Table 2 describes the sociodemographic characteristics of females and males without and with mask. First, we performed a descriptive analysis in which we established the differences between the participants by the mask use the intervention group with a one-way ANOVA. In this analysis, we can see in Table 3 that there are no significant variations between descriptive variables and the variables measured in the initial 3 ‘rest period' (in the baseline state) between the type of intervention (group A, B, or C) and mask use (wearing or not mask).

In addition, there were no significant differences in HR, SatO_2_, O_2_Hb, and HHb between the group using and not using the mask in the different intervention moments. Given that the previous analysis did not show significant differences between the mask use (vs. not wearing one). We analyzed the behaviour of each variable in the different phases of the intervention. In this analysis (see Table 4), we explored the HR, SatO_2_, O_2_Hb, and HHb behaviour during the seven intervention moments (baseline, WBV 1, rest 1, WBV 2, rest 2, WBV 3, and recovery).

The HR increased significantly in each period of exercise with WBV, while in the rest periods, it significantly decreases, although it does not reach basal values. SatO_2_ significantly decreases in periods of WBV exercise, while in periods of rest, it significantly increases again. There are no differences between baseline and the first rest; however, in the second rest and recovery (recovery), the SatO_2_ values increase after the intervention, with statistically significant differences. The O_2_Hb values follow the same pattern as those of SatO_2_. HHb significantly increases in periods of WBV exercise; while in periods of rest, it significantly decreases again. The first and second rest do not present any significant differences; however, HHb is significantly inferior in the baseline compared to the recovery. The HHb shows an inversed pattern to the ones in the SatO2 and O_2_Hb variables. All the reported values from Table 5 had a significance with a *p* < 0.001.

Considering the significant differences between HR, SatO_2_, O_2_Hb and HHb in the different moments, we calculated the effect size of these variants, comparing the other intervention moments with the baseline values. HR presents an increase between the rest period and WBV moments and an increase in the recovery period. The SatO_2_ shows a decrease in vibration moments and increase in recovery compared to the baseline moment. As for O_2_Hb, it happens the same as in SatO_2_, and the variations are practically the same in terms of numerical value. As for HHb, the opposite occurs with SatO_2_ and O_2_Hb; it increases in periods of vibration and decreases in recovery.

The analysis that compared the heart rate, muscle oxygen saturation, hemoglobin, oxyhemoglobin, and deoxyhemoglobin between the three groups in different intervention moments shows significant differences in all variables. The HR, in all groups, increases substantially from the baseline to WBV 1 and substantially declines from the WBV 2 to recovery. Group A presents a higher HR peak than the other groups in WBV 3, as the same happens to group B in WBV 3. Group C shows a less intense HR drop from rest 1/2 to WBV 1 and 2, less accentuated highs from rest 1 to WBV 2 and from rest 2 to WBV 3. The SatO_2_ and the O_2_Hb present the same pattern. Group C presents higher values during all moments, except for recovery than the other groups in these two variables. Group B shows a less accentuated declination of SatO_2_ and O_2_Hb from rest 2 to WBV 3 than the other groups. HHb follows an inverse trend to that seen in SatO2 and O2Hb. Group B shows a less accentuated increase of HHb from rest 2 to WBV 3 compared to group A and C (see Table 5).

## 4. Discussion

The present study is aimed at determining the acute effects of WBV exercise in the different phases of the intervention with the use of a mask vs. without a mask and different vibration frequency sequences. To determine the heart rate, muscle oxygen saturation, oxyhemoglobin, and deoxyhemoglobin differences, we established a brief WBV session protocol with three sets ( < 10 min), as it is a very common method to verify the acute effects of oxygen consumption using WBV [27]. The portable NIRS sensor was the same as used in the study by Vasquez-Bonilla [20], placed in the same place as in the previous study [10] to report changes in HR, blood pressure, and arterial oxygenation. A vibration load has been carried out during the ST, with a vibration frequency of 15 Hz and the feet were placed 25 cm apart; we might conclude that the monitoring of muscle oxygenation dynamics could be a valuable tool to evaluate performance [10].

Wearing a mask during exercise is a concern due to the possible impact it causes on performance; however, unlike previous studies that concluded that the use of masks in athletes causes hypoxic and hypercapnic breathing associated with a higher effort during exercise, our study shows that the use of surgical masks during the WBV has no influence on HR, SatO_2_, O_2_Hb, or HHb [21]. Supporting our results, there is a randomized crossover study that evaluated the effects of the cloth mask on arterial oxygen saturation (pulse oximetry) and vastus lateralis tissue oxygenation index (hemaglobin saturation/desaturation indicator) in 14 participants (using or not using a cloth mask) during an exhaustion cycle ergometry test [22].

Vibration produces exercise-related physiological changes in the variables measured; and in this study, we found differences between HR, SatO_2_, O_2_Hb, and HHb in WBV and periods without vibration stimulus. Similar results to our study were found with the use of WBV in the supine position on several physiological markers of physical activity, including muscle activation, oxygen consumption, and regional Hb oxygen saturation [28]. The values of preferred variables were higher during the vibration compared to the reference period and recovery period. In our study, the HR and the HHb increase during the WBV moments and the SatO_2_ and O_2_Hb present higher values when there is no vibrational stimulus. Showing a decrease in the level of muscle oxygenation when the WBV stimulus occurs due to muscle contraction, while during rest, the values recover almost to basal levels.

Conversely, the effects of the WBV on HR, SatO_2_, O_2_Hb, and HHb are sensitive to the different frequencies and the order that the frequencies are applied in the body. Thus, the effect of different WBV frequencies and amplitudes on oxygen consumption (VO_2_), carbohydrate rates, and fat oxidation was evaluated during treatment with WBV and subsequent aerobic exercise [29]. They applied seven vibration protocols, including one without WBV and six WBV: at 30 Hz/low amplitude (30 L), 30 Hz/high amplitude (30H), 40 Hz/low amplitude (40 L), 40 Hz/high amplitude (40H), 50 Hz/low amplitude (50 L), and 50 Hz/high amplitude (50H). They discovered that WBV combined with bodyweight squats could augment VO_2_ but not in all vibratory loads [29]. In our study, we used three different vibration intensities, using low (8hz), medium (12.5hz), and high (20hz) frequencies combined in 3 different interventions (A) 8, 12.6, and 20 Hz; (B) 12.6, 20, and 8 Hz; and (C) 20, 8, and 12.6 Hz. We were able to detect differences between the intervention groups in the different variables. In all groups, the higher HR peak was on the 20 Hz intervention. But group C shows a less intense HR drop from rest 1/2 to WBV 1 and 2, indicating that an 8 Hz stimulation after a 20 Hz, might not be enough to recruit oxygen. Concerning SatO_2_ and O_2_Hb, group C presents higher values during all moments, except for recovery, showing that this intervention is more exigent than others. Group B shows a smaller decline of SatO_2,_ O_2_Hb, and HHb values from rest 2 to WBV 3 showing that one minute of recovery might not be enough to recover basal values after a 20 Hz stimulation. Demonstrating the need for further research on the effect of different frequency patterns to obtain the best results for each intervention. Our study is limited by the similarities and controversies of the studies deriving from the great variety of vibrating platforms. We found difficulties in the discussion, especially when we tried to compare the mechanical stimulus since many of the studies published in this area do not indicate the type (vertical or lateral/sinusoidal) or the intensity of the applied vibration.

To pursue new research in WBV, we recommend analysing variations of the different variables in each minute of the recovery period. In addition to this research line, it might be helpful to implement and analyze new WBV protocols in neuromuscular pathology associated with muscle deoxygenation. The investigation in WBV can also benefit from further examination of the influence of different types of masks in several oxygen-related variables.

## 5. Conclusion

In conclusion, the present study demonstrated that HR, SatO_2_, and Hb are not influenced by the use of a surgical mask during WBV exercise. Generally, the WBV produces an increase in HR and HHb, and a decrease in SatO_2_ and O_2_Hb compared to vibration-free moments, despite these variables being sensitive to the different order of vibration frequencies during the intervention. Therefore, exercise on the vibrating platform can be an effective stimulus to improve tissue oxygenation values, adaptable to different necessities using different vibration frequency combinations.

## Figures and Tables

**Table 1:** Moments of the intervention plan (time and frequency relation).

Group		Baseline	WBV 1	Rest 1	WBV 2	Rest 2	WBV 3	Recovery
(A) 8_12_20	T (min)	3	1	1	1	1	1	3
	F (Hz)	—	8	—	12.6	—	20	—
(B) 12_20_8	T (min)	3	1	1	1	1	1	3
	F (Hz)	—	12.6	—	20	—	8	—
(C) 20_8_12	T (min)	3	1	1	1	1	1	3
	F (Hz)	—	20	—	8	—	12.6	—

T: time; min: minutes; F: frequency; Hz: hertz; WBV: whole-body vibration. (A) refers to the training group, WBV 1 = 8 Hz, WBV 2 = 12.6 Hz, and WBV 3 = 20 Hz; (B) refers to the training group, WBV 1 = 12.6 Hz, WBV 2 = 20 Hz, and WBV 3 = 8 Hz; (C) refers to the training group, WBV 1 = 20 Hz, WBV 2 = 8 Hz, and WBV 3 = 12.6 Hz.

**Table 2:** Sociodemographic characteristics of females and males without and with mask.

	No mask *n* = 20	Mask *n* = 27	*p*
Weight (kg)^a^	70.2 ± 11.1	66.1 ± 11.8	0.239
Height (m)^a^	1.74 ± 0.10	1.69 ± 0.10	0.091
BMI (kg/m^2^)^a^	22.9 ± 2.3	23.0 ± 2.4	0.986
Age (years)^a^	19.8 ± 1.9	20.0 ± 2.0	0.589
Gender^b^			0.029
Female	5 (25%)	11 (40.7%)	
Male	15 (75%)	16 (59.3%)	

^a^Values expressed as mean ± S.D., *p* values of one-way ANOVA. ^b^Values expressed as *n* (%), *p* values of analysis of chi-square test.

**Table 3:** Sample descriptive and baseline variables analyzes with one-way ANOVA by mask use and by group intervention.

			*n*	Mean		SD	*F*	*p*
FC (ppm)		*Total*	47	75.8	±	13.9		
	*Mask use*	No mask	20	75.3	±	14.2	0.08	0.780
		Mask	27	76.5	±	13.9		
	*Group intervention*	A	15	74.2	±	15.1	0.20	0.822
		B	15	77.5	±	16.2		
		C	17	75.8	±	11.2		
SatO_2_ (%)		*Total*	47	62.2	±	11.4		
	*Mask use*	No mask	20	62.7	±	11.6	0.11	0.740
		Mask	27	61.5	±	11.5		
	*Group intervention*	A	15	64.1	±	12.4	2.09	0.136
		B	15	57.3	±	11.4		
		C	17	64.8	±	9.6		
O_2_Hb (g/dl)		*Total*	47	7.6	±	1.3		
	*Mask use*	No mask	20	7.7	±	1.3	0.14	0.711
		Mask	27	7.5	±	1.3		
	*Group intervention*	A	15	7.8	±	1.4	2.12	0.132
		B	15	7.1	±	1.4		
		C	17	7.9	±	1.0		
HHb (g/dl)		*Total*	47	4.6	±	1.4		
	*Mask use*	No mask	20	4.6	±	1.4	0.11	0.740
		Mask	27	4.7	±	1.4		
	*Group intervention*	A	15	4.4	±	1.5	1.971	0.151
		B	15	5.2	±	1.4		
		C	17	4.3	±	1.2		

HR: heart rate, SatO_2_: muscle oxygen saturation, O_2_Hb: oxyhemoglobin, HHb: deoxyhemoglobin, N: sample, F: ANOVA variation between sample means, *p*: *p* value, and SD: standard deviation.

**Table 4:** ANOVA for repetitive measures (heart rate, muscle oxygen saturation, oxyhemoglobin, and deoxyhemoglobin) between moments.

	Baseline			WBV 1			Rest 1			WBV 2			Rest 2			WBV 3			Recovery			*F*	*p*	Post hoc
	Mean		SD	Mean		SD	Mean		SD	Mean		SD	Mean		SD	Mean		SD	Mean		SD			
HR (bpm)	75.8	±	14.0	112.8	±	16.9	96.3	±	15.5	111.9	±	18.7	100.7	±	19.1	114.3	±	20.1	89.4	±	18.2	166.6	<0.001	a, b, c, d, e, f, g, i, k, l, n, o, p, r, s, t, u

SatO_2_ (%)	62.2	±	11.5	44.4	±	16.5	61.7	±	10.3	49.3	±	17.8	65.1	±	10.9	52.9	±	20.6	71.2	±	10.7	45.3	<0.001	a, c, e, f, g, i, j, k, l, n, o, p, r, s, u

O_2_Hb (g/dL)	7.7	±	1.3	5.4	±	1.9	7.6	±	1.1	6.0	±	2.0	8.0	±	1.2	6.6	±	2.2	8.7	±	1.1	48.4	<0.001	a, c, e, f, g, i, j, k, l, n, o, p, r, s, u

HHb (g/dL)	4.7	±	1.4	6.8	±	2.1	4.7	±	1.3	6.3	±	2.2	4.3	±	1.4	5.6	±	2.4	3.5	±	1.4	47.8	<0.001	a, c, e, f, g, i, j, k, l, n, o, p, r, s, t, and u

a = baseline–WBV 1; b = baseline–rest 1; c = baseline–WBV 2; d = baseline–rest 2; e = baseline–WBV 3; f = baseline–recovery; g = WBV 1–rest 1; h = WBV 1–WBV 2; i = WBV 1–rest 2; j = WBV 1–WBV 3; k = WBV 1–recovery; l = rest 1–WBV 2; m = rest 1–rest 2; n = rest 1–WBV 3; o = rest 1–recovery; p = WBV 2–rest 2; q = WBV 2–WBV 3; r = WBV 2–recovery; s = rest 2–WBV 3; t = rest 2–recovery; u = WBV 3–recovery; HR: heart rate; SatO_2_: muscle oxygen saturation; O_2_Hb: oxyhemoglobin; HHb: deoxyhemoglobina; F: degrees of freedom; and *p*: *p* value.

**Table 5:** Baseline, training, rest, and recovery data of the three training groups for heart rate, muscle oxygen saturation, hemoglobin, oxyhemoglobin, and deoxyhemoglobin.

		Baseline			WBV 1			Rest 1			WBV 2			Rest 2			WBV 3			Recovery			*p* values	Pairwise comparisons
HR (bpm)	(A)	74.3	±	15.0	107.0	±	17.0	91.4	±	13.0	107.0	±	15.0	95.2	±	17.0	123.0	±	19.0	92.4	±	19.1	<0.001	a, b, c, d, e, f, g, i, j, k, l, n, p, q, r, s, and u
	(B)	77.5	±	16.0	122.0	±	17.0	94.1	±	17.0	123.0	±	17.0	108.0	±	21.0	110.0	±	21.0	88.3	±	20.6	0.002	a, b, c, d, e, f, g, h, k, l, m, n, o, p, q, r, t, and u
	(C)	75.9	±	11.0	119.0	±	16.0	103.0	±	20.0	106.0	±	20.0	98.8	±	19.0	111.0	±	10.0	87.9	±	15.8	<0.001	a, b, c, d, e, f, g, h, i, k, o, p, r, s, t, and u

SmO_2_ (%)	(A)	64.1	±	12.0	41.6	±	16.0	60.2	±	9.4	43.7	±	17.0	62.8	±	10.0	43.2	±	21.0	71.5	±	9.3	0.034	a, c, e, f, g, i, k, l, n, o, p, r, s, t, and u
	(B)	57.4	±	12.0	44.7	±	15.0	59.3	±	9.7	48.2	±	13.0	62.6	±	9.4	61.6	±	14.0	71.8	±	11.1	0.001	g, i, j, k, l, o, q, r, s, and u
	(C)	64.8	±	9.6	46.8	±	18.0	65.2	±	11.0	55.3	±	21.0	69.3	±	12.0	53.8	±	23.0	70.6	±	12.2	0.021	a, g, k, l, m, o, p, q, r, s, and u

Hb (g/dL)	(A)	12.3	±	0.2	12.3	±	0.3	12.3	±	0.3	12.3	±	0.3	12.3	±	0.3	12.6	±	0.8	12.3	±	0.2	0.222	—
	(B)	12.4	±	0.5	12.4	±	0.5	12.4	±	0.5	12.3	±	0.5	12.3	±	0.5	12.3	±	0.5	12.3	±	0.5	0.450	—
	(C)	12.4	±	0.5	12.4	±	0.6	12.4	±	0.5	12.5	±	0.5	12.4	±	0.4	12.4	±	0.5	12.4	±	0.4	0.419	—

O_2_Hb (g/dL)	(A)	7.9	±	1.5	5.1	±	1.9	7.4	±	1.1	5.3	±	2.0	7.7	±	1.2	5.7	±	2.1	8.8	±	1.1	0.003	a, c, e, f, g, i, k, l, o, p, r, s, t, and u
	(B)	7.1	±	1.4	5.5	±	1.7	7.3	±	1.1	5.9	±	1.5	7.7	±	1.1	7.5	±	1.6	8.8	±	1.1	0.001	g, i, j, k, l, o, p, q, r, s, and u
	(C)	8.0	±	1.0	5.7	±	2.2	8.0	±	1.1	6.8	±	2.4	8.6	±	1.3	6.6	±	2.6	8.7	±	1.3	0.022	a, g, i, k, m, o, p, r, s, and u

HHb (g/dL)	(A)	4.4	±	1.6	7.2	±	2.0	4.9	±	1.2	6.9	±	2.2	4.6	±	1.3	6.4	±	2.2	3.5	±	1.2	0.001	a, c, e, f, g, i, k, l, o, p, r, t, and u
	(B)	5.3	±	1.4	6.8	±	2.0	5.1	±	1.2	6.4	±	1.7	4.6	±	1.2	4.8	±	1.9	3.5	±	1.5	0.001	a, g, i, j, k, l, o, p, q, r, t, and u
	(C)	4.4	±	1.3	6.6	±	2.5	4.4	±	1.5	5.6	±	2.7	3.8	±	1.6	5.8	±	2.9	3.7	±	1.6	0.021	a, g, i, l, m, o, p, r, s, and u

P values of repeated measures ANOVA. Pairwise comparisons *p* < 0.05: a = baseline–WBV 1; b = baseline–rest 1; c = baseline–WBV 2; d = baseline–rest 2; e = baseline–WBV 3; f = baseline–recovery; g = WBV 1–rest 1; h = WBV 1–WBV 2; i = WBV 1–rest 2; j = WBV 1–WBV 3; k = WBV 1–recovery; l = rest 1–WBV 2; m = rest 1–rest 2; n = rest 1–WBV 3; o = rest 1–recovery; p = WBV 2–rest 2; q = WBV 2–WBV 3; r = WBV 2–recovery; s = rest 2–WBV 3; t = rest 2–recovery; and u = WBV 3–recovery. HR: heart rate; SmO_2_: muscle oxygen saturation; Hb: hemoglobin; O_2_Hb: oxyhemoglobin; HHb: deoxyhemoglobin; WBV: whole-body vibration; (A) = training group WBV 1 = 8 Hz, WBV 2 = 12 Hz, and WBV 3 = 20 Hz; (B) = training group WBV 1 = 12 Hz, WBV 2 = 20 Hz, and WBV 3 = 8 Hz; and (C) = training group WBV 1 = 20 Hz, WBV 2 = 8 Hz, and WBV 3 = 12 Hz.

## Data Availability

The data supporting this systematic review are from previously reported studies and datasets, which have been cited.
